# Cell adhesion-induced transient interaction of ADAM15 with poly(A) binding protein at the cell membrane colocalizes with mRNA translation

**DOI:** 10.1371/journal.pone.0203847

**Published:** 2018-09-28

**Authors:** Beate B. Böhm, Yuliya Fehrl, Tomasz Janczi, Nadine Schneider, Harald Burkhardt

**Affiliations:** 1 Division of Rheumatology, University Hospital Frankfurt, Goethe University, Frankfurt am Main, Germany; 2 Project Group Translational Medicine & Pharmacology TMP, Fraunhofer Institute for Molecular Biology and Applied Ecology IME, Frankfurt am Main, Germany; Technische Universitat Dresden, GERMANY

## Abstract

The regulation of temporo-spatial compartmentalization of protein synthesis is of crucial importance for a variety of physiologic cellular functions. Here, we demonstrate that the cell membrane-anchored disintegrin metalloproteinase ADAM15, upregulated in a variety of aggressively growing tumor cells, in the hyperproliferative synovial membrane of inflamed joints as well as in osteoarthritic chondrocytes, transiently binds to poly(A) binding protein 1 (PABP) in cells undergoing adhesion. The cytoplasmic domain of ADAM15 was shown to selectively interact with the proline-rich linker of PABP. Immunostainings of adhesion-triggered cells demonstrate an ADAM15-dependent recruitment of PABP to cell membrane foci coinciding with ongoing mRNA translation as visualized by the detection of puromycin-terminated polypeptides. Moreover, the increase in cell membrane-associated neosynthesis of puromycylated proteins upon induction of cell adhesion was proven linked to ADAM15 expression in HeLa and ADAM15-transfected chondrocytic cells. Thus, down regulation of ADAM15 by siRNA and/or the use of a cell line transfected with a mutant ADAM15-construct lacking the cytoplasmic tail resulted in a considerable reduction in the amount of cell membrane-associated puromycylated proteins formed during induced cell adhesion.

These results provide first direct evidence for a regulatory role of ADAM15 on mRNA translation at the cell membrane that transiently emerges in response to triggering cell adhesion and might have potential implications under pathologic conditions of matrix remodeling associated with ADAM15 upregulation.

## Introduction

ADAM15 belongs to the family of ADAMs (a disintegrin and metalloproteinase) and is a transmembrane protein, with its larger extracellular part being organized in distinct functional domains, a prodomain, a metalloproteinase domain, a disintegrin and a cysteine-rich domain, followed by a transmembrane and a cytoplasmic tail of 100 amino acids [1]. ADAM15 plays a role in cell-cell communication and cell-matrix interaction via binding of its RGD consensus motif containing disintegrin domain to various integrin α and β chains [2, 3]. Due to its involvement in cell adhesion ADAM15 plays a role in neovascularization and angiogenesis, processes that are tightly associated with chronic inflammation [4]. It is highly upregulated in the inflamed synovial membrane of patients with osteoarthritis (OA) and rheumatoid arthritis (RA) [5] and an accelerated development of murine osteoarthritis in ADAM15 knockout mice suggested a homeostatic rather than a destructive role of ADAM15 in cartilage remodeling [6]. Besides its function as a cell adhesive protein ADAM15 is also implicated in anti-apoptotic pathways that render human chondrocytes more resistant to genotoxic stress by upregulating the X-linked inhibitor of apoptosis (XIAP) [7]. Additionally, ADAM15 contributes to apoptosis-resistance of RA synovial fibroblasts by enhancing phosphorylation of focal adhesion kinase (FAK) and c-src kinase upon triggering Fas/CD95, a death receptor belonging to the tumor necrosis factor receptor superfamily [8].

Furthermore, a significantly upregulated ADAM15 expression is detected in various solid tumors, e.g. breast and prostate, pancreas, lung and colon carcinomas [9–11] and its correlation with cancer progression and metastasis is associated with strong overexpression of ADAM15 as well as an increased migratory capacity of the tumor cells [12, 13].

Poly(A) binding protein (PABP), a highly conserved cytoplasmic protein, plays a critical role in mRNA translation and stability by binding to the 3’ poly(A) tail of eukaryotic mRNAs [14]. Its structure is composed of a highly conserved N-terminus containing four tandem RNA recognition motifs (RRM) and a C-terminus that harbors the proline-rich linker and the PABC domain. The first two RRMs are sufficient for specific poly(A) binding [15] and RRM4 is responsible for most of the nonspecific RNA binding of PABP [14]. PABP plays a key role as a translation initiation factor and its interaction with the elongation initiation factor 4G (eIF4G) mediates circularization of the mRNA, by linking the 5’ cap and the 3’ poly(A) tail in a closed loop structure, thereby stimulating translation of fully processed, intact mRNAs [16]. PABP stimulates ribosome recruitment to the mRNA both at the 40S ribosome subunit recruitment and 60S subunit joining steps [17].

The C-terminal domain of PABP (PABC) spans the last 80 amino acids and is arranged in 5 α-helices [14]. Several proteins from the translation machinery as well as translational control, e.g. the translation termination factor eRF3, eIF4B, and PABP interacting protein 1 and 2 (Paip1 and Paip2) can bind to this domain [18–20]. The C-terminus can contribute to mRNA stabilization and also plays a role in the nuclear export of PABP bound to newly synthesized poly(A) containing RNA [21]. A proline-rich linker connects the PABC domain to the RRM cluster and is responsible for multimerization of PABP and its cooperative binding to poly(A) [22, 23]. The linker contains proteolytic cleavage sites for proteases of a wide range of viruses affecting the activity of PABP, its stability and intracellular localization during viral infections [24].

In this study, we describe a novel interaction between ADAM15 and PABP, which was initially identified by MALDI-TOF in ADAM15 immunoprecipitations. Mammalian-two hybrid and protein binding studies using various recombinant PABP domains and the cytoplasmic region of ADAM15 revealed the proline-rich linker of PABP as being critical for ligation with ADAM15. However, the newly uncovered protein interaction seems to be tightly regulated spatio-temporally as a colocalization of both proteins at the plasma membrane remained visually detectable only for the process during which the cells were undergoing adhesion. Validation of these findings by immunodetection in cell surface biotinylated membrane preparations revealed a continuous increase of PABP in the membrane fractions during the adhesion process, which was critically dependent on ADAM15. Also, the adhesion-induced colocalization of ADAM15 and PABP coincides with an ongoing protein synthesis in situ, as immunodetection of puromycin terminated proteins of cells fixed during adhesion revealed colocalization with ADAM15 and PABP, respectively, at the cell membrane.

In conclusion, our studies demonstrate that ADAM15 and PABP tether at the cell membrane upon triggered cell adhesion, which colocalizes with an ongoing mRNA translation.

## Results

### The cytoplasmic tail of ADAM15 interacts with poly(A) binding protein 1 (PABP)

**Fig 1:**
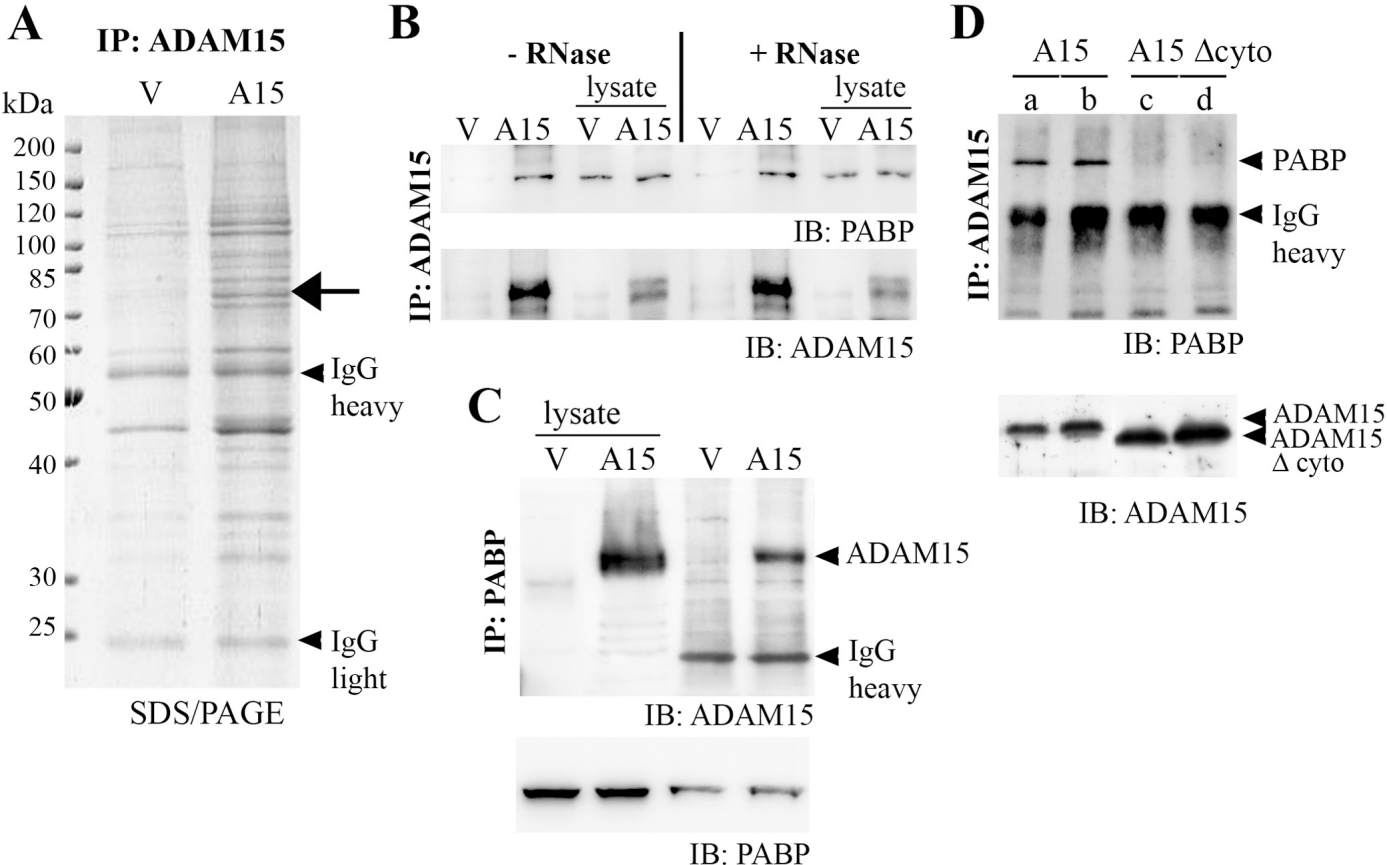
ADAM15 co-immunoprecipitates with poly(A) binding protein 1 (PABP). (A) Co-immunoprecipitations (IP) of chondrocyte lysates transfected with a vector control (V) or full-length ADAM15 (A15) using anti-ADAM15 antibodies were separated by SDS/PAGE. A specifically precipitated protein at 75 kDa in ADAM15- cells was identified as poly(A) binding protein 1 (arrow). (B, C) IPs using anti-ADAM15 or anti-PABP antibodies of V- and ADAM15-transfected cells and subsequent immunoblot (IB) using anti-PABP or vice versa ADAM15 antibodies confirmed a specific precipitation of PABP in the ADAM15-transfected cells only. As controls, the blots were reprobed with ADAM15 or PABP antibodies, respectively. (B) IPs of cell lysates incubated with in lysis buffer ± RNase I (5 U/ml) overnight using anti-ADAM15 antibodies, showing ADAM15/PABP binding in the ADAM15-transfected cells only, which is independent of mRNA potentially bound to PABP. (D) IPs of A15-lysates and a deletion mutant of ADAM15 lacking the cytoplasmic tail (A15 Δcyto) using 2 different anti-ADAM15 antibodies (a, c, mouse and b, d, goat) specifically precipitates PABP in lysates that expressed full-length ADAM15 only. As control, the blot was reprobed using anti-ADAM15 antibodies, showing full-length ADAM15 (~100 kDa) and A15 Δcyto (~ 90 kDa).

To analyze proteins that interact with the cytoplasmic domain of ADAM15 co-immunoprecipitation was employed. The chondrocyte cell line T/C28a4 stably transfected with full-length ADAM15 or an empty vector control were immunoprecipitated using anti-ADAM15 antibodies and analyzed by SDS/PAGE (Fig 1A). A prominent protein band at ~ 75 kDa was excised and subjected to peptide mass fingerprinting and mass spectrometry (MALDI-TOF). The protein was identified as poly(A) binding protein 1 (PABP). Subsequent immunoprecipitation (IPs) of cell lysates from full-length ADAM15-transfected cells using anti-ADAM15 antibodies and immunoblotting using anti-PABP antibodies confirmed a specific co-precipitation of ADAM15 with PABP as compared to IPs of vector-transfected cells (Fig 1B). Vice versa, IPs using anti-PABP antibodies and immunodetection of ADAM15 also detected co-precipitation of PABP and ADAM15 in ADAM15-cells only (Fig 1C). Also, to exclude whether the ADAM15/PABP binding is mediated by mRNAs still bound to PABP in the lysate, pretreatment of cell lysates with RNase I (5 U/ml) and subsequent immunoprecipitation overnight clearly revealed an interaction of ADAM15 with PABP that is independent of the presence of mRNA potentially bound to PABP (Fig 1B). Moreover, binding of ADAM15 to PABP was clearly dependent on the intracellular domain of ADAM15, since PABP could not be precipitated using both goat and mouse anti-ADAM15 antibodies in cell lysates expressing a cell surface ADAM15-mutant that lacks the cytoplasmic tail (ΔC) as compared to cells transfected with the full-length ADAM15 (Fig 1D). The Western Blot analyses as well as FACS analysis of the chondrocyte cell line transfected with full-length and the cytoplasmic deletion mutant of ADAM15 have been described in detail earlier [25].

### ADAM15 binds to the proline-rich domain of PABP

#### Protein binding assays using recombinant fragments

**Fig 2:**
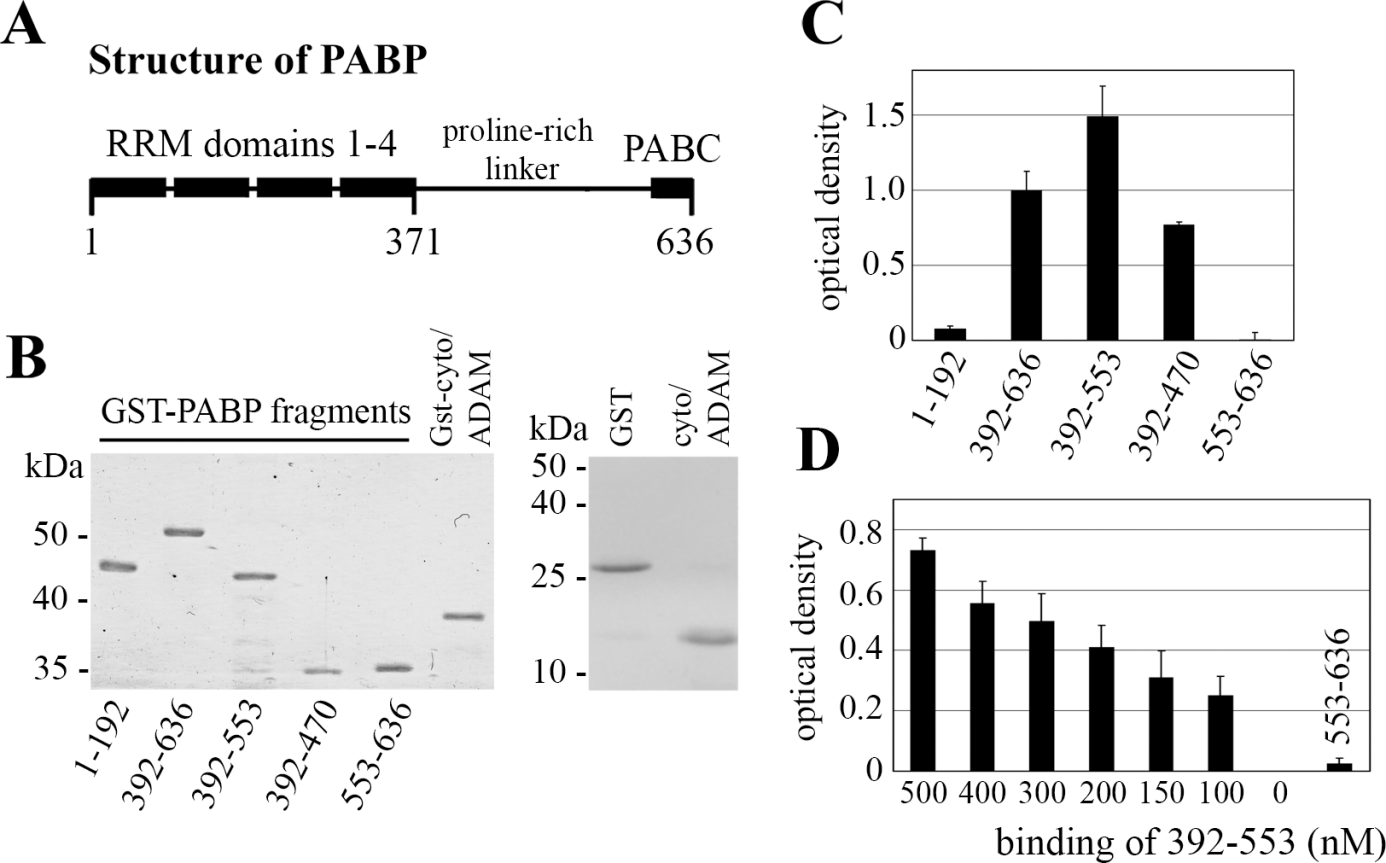
ADAM15 binds to the proline-rich domain of PABP. (A) Domain structure of PABP. (B) left, SDS/PAGE (10%) of recombinant expressed GST-tagged PABP domains and the cytoplasmic domain of ADAM15; right, SDS/PAGE (14%) of the purified cytoplasmic domain of ADAM15 and GST protein. (C) binding of the various GST-tagged PABP domains to cytoplasmic domain of ADAM15 immobilized to a 96-well, showing strongest binding of the proline-rich domain 392–553, and no binding to the RRM domain 1–192 and the C-terminal PABC domain 553–636. (D) binding of PABP fragment 392–553 in concentrations from 0–500 nM to immobilized ADAM15 revealed still binding above an OD of 0.2 in low nM concentration (100nM), as compared to the binding of PABP 553–663, which showed no binding at a concentration of 500 nM.

To determine the domains of PABP that bind to the cytoplasmic tail of ADAM15, protein binding assays in ELISA format using recombinant protein fragments were employed. GST-tagged PABP protein fragments from the N-terminus (RRM1 and 2: 1–192), the linker domain (392–553, 392–470), the C-terminal PABC domain (553–636), and the complete C-terminus (392–636) as well as the GST-tagged cytoplasmic domain of ADAM15 were recombinantly expressed in BL21 E. coli and the purified proteins analyzed by SDS/PAGE exhibited electrophoretic mobilities corresponding to the respectively calculated molecular weights (Fig 2B). The GST-tag from the cytoplasmic ADAM15 fragment was removed by prescission protease and yields a protein of ~ 15 kDa (Fig 2B). The cloning and purification of the cytoplasmic domain of ADAM15 has also been described earlier in detail [25].

The recombinant ADAM15 cytoplasmic domain (cytoADAM) was bound to ELISA plates overnight, followed by binding of the various GST-tagged PABP domains and detection using anti-GST antibodies. The binding assays revealed that the C-terminus of PABP (392–636) that comprises the proline-rich and the PABC domain (Fig 2A) binds to cytoADAM, whereas no binding of the RNA binding domains 1 and 2 (RRM, 1–192) as well as the C-terminal PABC domain (553–636) was detected (Fig 2C). Upon deletion of the PABC domain (553–636) the proline-rich domain (392–553) exhibited a somewhat stronger binding to cytoADAM, suggesting that the absence of the C-terminus favours a PABP conformation with improved cytoADAM binding properties. However, a further C-terminal shortening by 83 amino acids (392–470) reduced the binding to cytoADAM by ~ 50% (Fig 2C), suggesting the integrity of the complete proline-rich domain is necessary for optimal binding to ADAM15.

Furthermore, the concentration dependency of the binding of the proline-rich fragment 392–553 to immobilized cytoADAM15 was analyzed in a range from 0–500 nM under the same experimental conditions as described above. As the fragment at a concentration of a 100 nM still yielded an OD above > 0.2 in the binding assay, a considerably high affinity interaction with the cytoplasmic ADAM15 domain in the nanomolar range is suggested (Fig 2D).

#### Protein binding studies using mammalian-two hybrid

**Fig 3:**
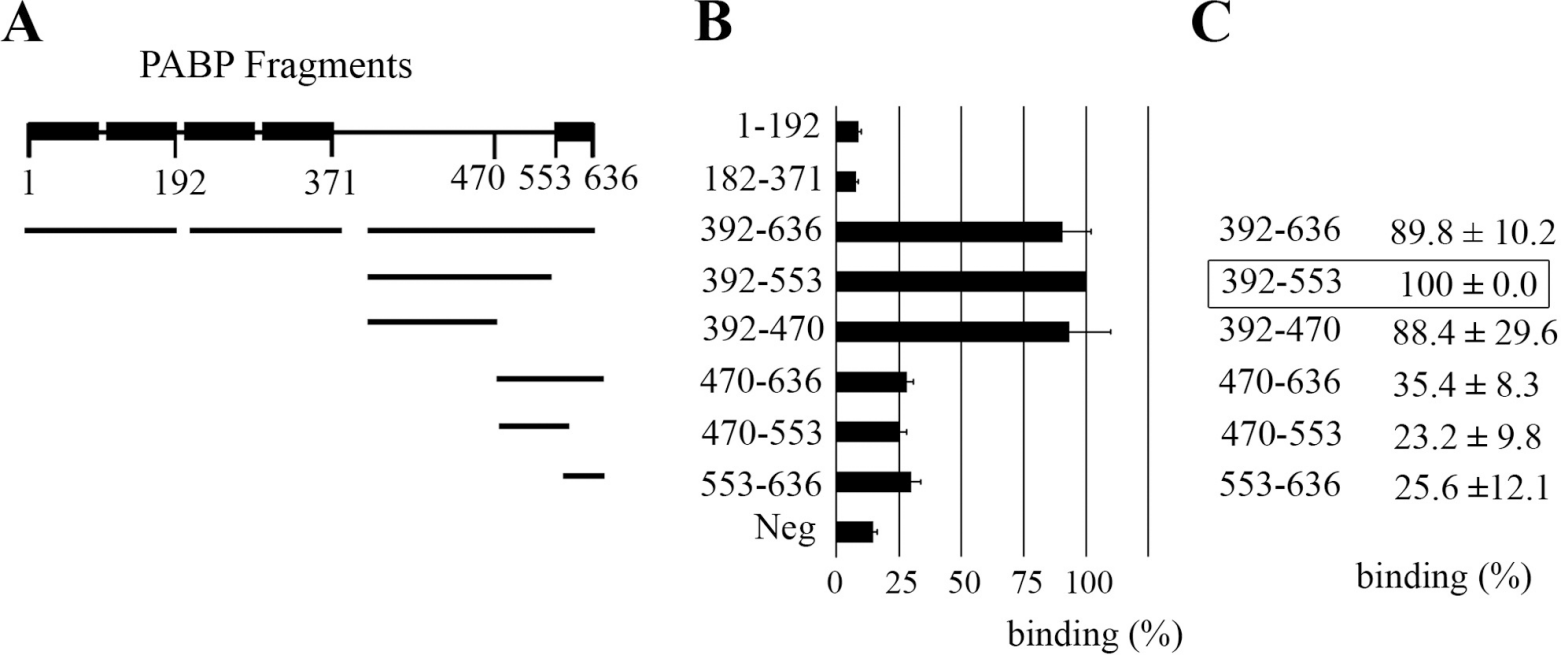
ADAM15 binds directly to the proline-rich linker of PABP. Mammalian-two hybrid was used for the identification of the PABP domain interacting with ADAM15. (A) PABP fragments covering the complete sequence were cloned into a bait vector, the cytoplasmic domain of ADAM15 into a prey vector and co-transfected together with a firefly luciferase reporter into HEK293 cells, and transcribed luciferase signals measured after 48 hours. (B) An exclusive binding of ADAM15 to the proline-rich domain of PABP (392–470), but not the RRM domains 1 and 2 (1–192) as compared to an included negative protein binding control (p53 and TRAF). A representative experiment is shown. (C) depicts the mean (± S.D.) of five repeated assays of ADAM15-binding with each distinct PABP fragment. Measured luciferase values were normalized to the values obtained from a co-transfected Renilla luciferase control plasmid.

To further substantiate these findings in an independent experimental approach, mammalian-two hybrid technology was applied for analyzing the interaction of ADAM15 with PABP, allowing not only fine-mapping of protein binding domains, but also the detection of a direct binding of two proteins. cDNA of protein fragments, covering the whole PABP sequence (Fig 3A), were cloned into a bait vector that contains the DNA-binding domain of GAL4 (galactose-responsive transcription factor), thus resulting in the expression of a GAL4-/PABP fusion protein after transfection in mammalian cells. Accordingly, the cytoplasmic domain of ADAM15 was cloned into the prey vector that contains the NFκB transactivation domain, as described in detail [25], expressing an NFκB/cytoADAM fusion protein. Measurable luminescence signals resulting from the transcribed luciferase served as a quantitative indicator for protein interaction that is generated when the two interacting proteins tether the NFκB and GAL4 domains to the GAL4 elements of the luciferase promotor. The various PABP domains in the bait vector, the cytoplasmic domain of ADAM15 in prey vector were then co-transfected together with firefly luciferase reporter and a Renilla control plasmid into HEK cells.

ADAM15 exhibited strong binding to the C-terminus of PABP 392–636, which was slightly stronger when the PABC domain (553–636) was removed (Fig 3B), mirroring the protein binding studies, where the recombinant protein fragment (392–553) also exhibited the strongest binding to ADAM15 (Fig 3B). Therefore, the binding of ADAM15 to PABP fragment (392–553) was set to 100%. Further size reduction by 83 amino acids (392–470) still demonstrated a strong binding of 88.4 ± 29.6% of the predefined maximum, when calculated from 5 independently performed experiments (Fig 3C), albeit the higher standard deviation might reflect a slightly increased instability of the expressed polypeptide. Virtually no binding of the RRM domains 1–4 (1–192 and 182–371) to ADAM15 was observed as compared to an included negative protein binding control (p53 and TRAF). Also, the binding of ADAM15 to either of the three protein fragments of the C-terminus of PABP (470–636, 470–553 or 553–636) exhibited very low levels of luciferase induction, remaining only slightly elevated above the range of the negative control. Taken together, all protein binding studies revealed that ADAM15 binding is confined to the proline-rich linker domain of PABP between amino acids 392–553.

### ADAM15 colocalizes transiently with PABP at the cell surface upon triggering cell adhesion

#### Colocalization studies using double immunofluorescence stainings

**Fig 4:**
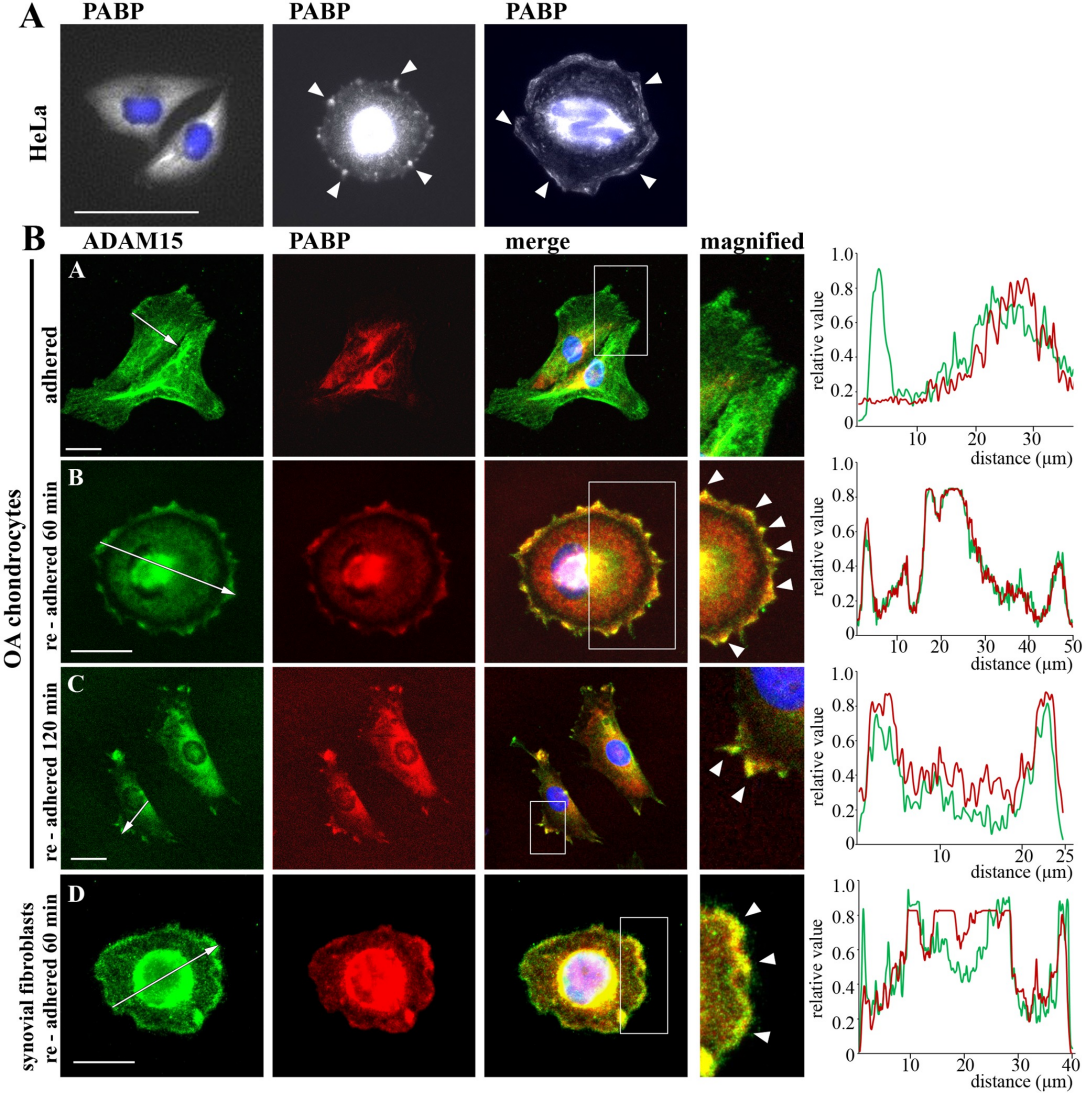
PABP transiently colocalizes with ADAM15 at the cell surface after re-adhesion. (A) Staining of HeLa cells with anti-PABP and Alexa 488 antibodies, showing a cytoplasmic staining in the majority of cells (left), rounded cells (middle) or cells during cell division (right) also reveal a dot-like staining of PABP at the cell surface (white arrows). (B) Confocal microscopy of double stainings of osteoarthritic (OA) chondrocytes and synovial fibroblasts using anti-PABP and ADAM15 antibodies and visualization with Alexa 488 (green) and Alexa 594 (red) secondary antibodies, showing cytoplasmic staining of PABP and membrane staining of ADAM15 in normally grown, adherent cells. After detachment and re-adhesion of cells to a chamberslide for 60 min redistribution of PABP to the cell surface was detected, which colocalized with ADAM15 (merge in yellow) along the entire cell membrane and was drastically reduced in cells already spread after 120 min of adhesion (white arrows). White inset shows a magnified area, objective 40x, size bar = 20 μm. Quantification of the pixel density of the ADAM15/PABP (co-) localization. The graph represents the signal intensity of ADAM15 (green) and PABP (red) following the cell axis (indicated by a white arrow) of the cells adjacent to the graph.

To elucidate the subcellular localization of ADAM15 and PABP double immunofluorescence stainings using anti ADAM15 and PABP antibodies were employed. Upon testing the staining condition for the PABP antibody on HeLa cells, as expected, the majority of the cells displayed a homogenous localization of PABP in the cytoplasm, and no staining of PABP in the nucleus (Fig 4A, left). However, about 20% of cells that displayed a round shape or laying closely together, presumably in the process of cell division, displayed—besides a strong staining around the nucleus—also a dot-like staining pattern for PABP at the cell surface. This peripheral dot-like PABP staining pattern was characteristic for rounded up cells that tended to flatten into dash-like structures in more spread cells (Fig 4A, middle and right). Double immunofluorescence stainings of primary chondrocytes and synovial fibroblasts grown as adherent cells for ADAM15 and PABP show that ADAM15 is immunodetected both in the cytoplasm and at the cell surface, whereas PABP remains confined to the cytoplasm within a more restricted area around the nucleus (Fig 4B, adhered). However, virtually no staining for PABP is detectable at the cell membrane under these conditions. To address the question, whether an interaction of the transmembrane anchored ADAM15 and the cytoplasmic PABP found in vitro can also occur under certain conditions in vivo, a “re-adhesion assay” was established, whereby OA chondrocytes or synovial fibroblasts grown on tissue culture dishes, were detached by EDTA and allowed to re-attach to a chamberslide for 60–120 minutes. The fixed cells double stained with anti-ADAM15 and PABP antibodies now revealed a completely different staining pattern after 60 min of adhesion. At this time point the cells were still round demonstrating the presence of PABP at the entire cell membrane in colocalization with ADAM15. Cells that were re-adhered for 120 min and already showing spreading still exhibit colocalization of both proteins in focal adhesion like structures to some degree, however its extent was drastically diminished as compared to the rounded cell state (Fig 4B). These results suggest that the interaction of PABP and ADAM15 at the cell membrane is confined to cells in the process of adhesion that fades upon completion.

#### Colocalization studies using cell surface biotinylation

**Fig 5:**
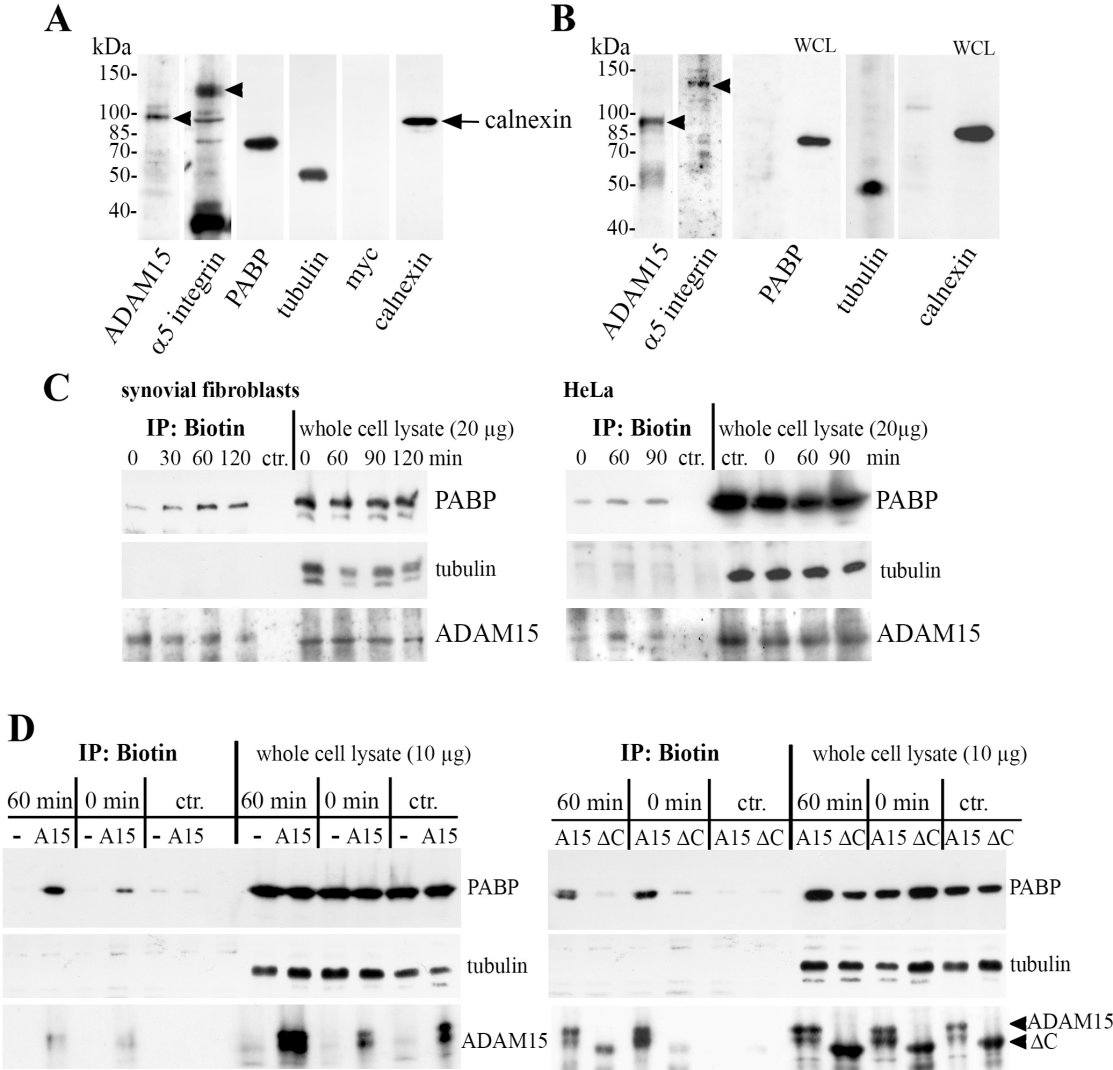
PABP redistributes to the cell membrane after re-adhesion in dependency of ADAM15. (A) Immunoblot of enriched plasma membrane fractions isolated by sequential (ultra-) centrifugation, showing ADAM15 and integrin α5 as membrane marker proteins, and also detection of PABP and tubulin as well as calnexin, a marker for ER membranes; myc, marker for nucleus, was negative. (B) Immunoblot of PFA-fixed (5% for 5 min), cell surface biotinylated plasma membrane proteins isolated using streptavidin-conjugated magnetic beads, detecting ADAM15 and integrin α5, but not calnexin and PABP. WCL = whole cell lysate. (C, D) Cells were re-adhered to a chamberslide for various time points, fixed with PFA (1% for 5 min), and cell surface biotinylated membrane proteins isolated by streptavidin magnetic beads as well as whole cell lysates prior to purification on the magnetic beads were subjected to immunoblotting using anti-PABP antibodies. Blots were reprobed using tubulin and ADAM15 antibodies as loading controls. (C) In isolated cell membranes of synovial fibroblasts and HeLa cells, detection of increased amounts of PABP at the plasma membrane upon adhesion for 30–120 min and (D, left panel) increased amount of PABP at the plasma membrane after 60 min adhesion in chondrocytes transfected with full-length ADAM15 (A15) in comparison to vector control-transfected cells (-) or (D, right panel) cells transfected with a deletion mutant of ADAM15 lacking the cytoplasmic tail (ΔC). Contr: non biotinylated cell lysates enriched on streptavidin magnetic beads served as a background control.

An independent experimental approach was employed to provide further evidence for the interaction of ADAM15 and PAPB at the cell surface using isolated cell membrane preparations and Western blotting. Initially, a classical purification of cell membranes by sequential centrifugation steps was performed and analyzed for purity by immunoblotting using antibodies against different marker proteins: ADAM15 and α5 integrin as a marker for cell membrane, tubulin, myc as a nuclear marker and calnexin as a marker for the endoplasmic reticulum (ER). Although the cell membrane preparation was virtually free of myc, huge amounts of calnexin could be detected, revealing the presence of ER membranes in the preparation (Fig 5A). Since it was mandatory, to get rid of any ER organelle proteins, and since both ADAM15 and PABP are also present in this compartment, a different isolation procedure of cell membranes was used. Cells grown on petri dishes were cell surface biotinylated and biotinylated proteins pulled down using streptavidin-coated magnetic beads. The immunoblot analysis showed the presence of ADAM15 and integrin α5, but was completely negative for the expression of PABP and calnexin (Fig 5B), thus for all subsequent experiments when applicable cell surface biotinylation and purification by streptavidin was used.

In order to study the redistribution of PABP to the cell surface upon stimulation, analogous to the immunostaining results, synovial fibroblasts and HeLa cells were detached with EDTA, re-adhered for 30–120 minutes, shortly fixed with paraformaldehyde (1% for 5 min) and cell surface biotinylated. The streptavidin affinity purified biotinylated cell surface proteins were analyzed for the presence of PABP by immunoblotting using anti-PABP antibodies. Both, synovial fibroblasts as well as HeLa cells display a recruitment of PABP with increasing detection intensities in the plasma membrane fractions, starting as early as 30 min after induction of adhesion with a maximum at 60 min (Fig 5C). These results clearly demonstrate the presence of PABP at the cell membrane, suggesting a redistribution of PABP to the cell surface in response to the re-adhesion stimulus. The cell membranes of both cell lines exhibited a high protein expression of ADAM15, which was not affected by the re-adhesion and also detected in the membrane of normally grown cells (0 min time point, Fig 5C). In parallel, same amounts of whole cell lysates before the purification step on streptavidin magnetic beads were analyzed for PABP that served as an additional control for an equal input of protein amount.

To further elucidate the dependence of PABP recruitment to the cell membrane on ADAM15 expression during adhesion, chondrocyte cell lines transfected with either full-length ADAM15 (A15) or a mutant consisting of the entire membrane-anchored extracellular region but lacking the cytoplasmic tail (ΔC) were studied. Cells were subjected to re-adhesion for 60 min, and the purified surface biotinylated protein analyzed by immunoblotting using anti-PABP antibodies. Membranes derived from cells transfected with the complete ADAM15 displayed a markedly enhanced signal for PABP after 60 min of re-adhesion compared with cells that were not re-adhered at 0 min (Fig 5D, left panel). In addition, cells transfected with an empty vector (-) did not display any signal for PABP in the membrane preparations, which was in the range of the background signal of non-biotinylated lysates precipitated with streptavidin conjugated magnetic beads that served as a negative control. Furthermore, cells transfected with the ADAM15-mutant lacking the cytoplasmic tail (ΔC) did not display any signal for PABP at the cell membrane (Fig 5D). Taken together, these results clearly show that the recruitment of PABP to the cell membrane during adhesion is critically dependent on the expression of the cytoplasmic domain of ADAM15.

### Protein translation is detected at the cell surface during adhesion

To address the question, whether the recruitment of PABP to the cell membrane also implicates an ongoing mRNA translation at the cell surface in areas of ADAM15 expression, the so-called SUnSET (Surface Sensing of Translation) assay, a nonradioactive method to monitor protein synthesis, was employed [26]. This method uses puromycin labeling of cells, which gets incorporated into nascent protein chains, thereby causing premature termination of protein translation. The subsequent immunodetection of terminated protein chains using anti-puromycin antibodies allows then detection of punctate, dot-like structures that represent spots of ongoing mRNA translation.

**Fig 6:**
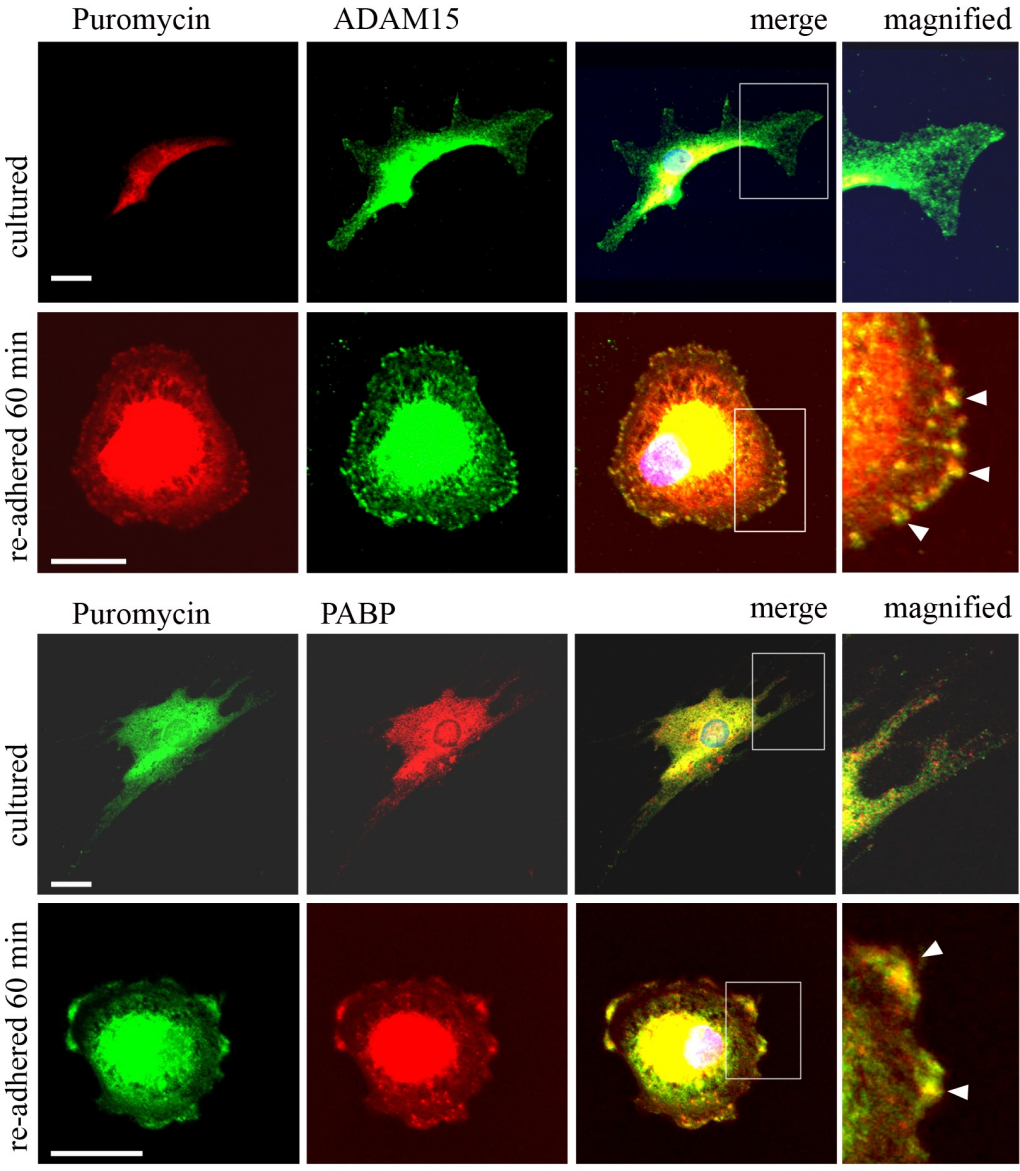
Puromycin terminated proteins colocalize with ADAM15 and/or PABP at the cell surface. Confocal microscopy of human osteoarthritic chondrocytes double stained with anti-puromycin and anti-ADAM15 antibodies and with anti-puromycin and anti-PABP antibodies and visualized using Alexa 488 (green) and Alexa 594 (red) secondary antibodies, showing co-localization of puromycin-conjugated proteins at the plasma membrane (white arrows, magnified inset) with ADAM15 and PABP after induction of adhesion for 60 min, which is not detected in normally cultured cells. White inset shows magnified area. Objective 40x, size bar = 20μm.

Primary human osteoarthritic chondrocytes and synovial fibroblasts that were either grown on chamberslides or re-adhered for 60 min were fed with puromycin and cycloheximide (100 μg/ml) to block the dissociation of polypeptide-puromycin conjugates from ribosomes, fixed with PFA and double stained for either ADAM15 and puromycin or PABP and puromycin. Both cell types when cultured overnight displayed a strong staining for puromycin-terminated proteins in the cytoplasm with a major positively stained area around the nucleus (Fig 6). Virtually no signals derived from puromycylated protein chains were detectable at the cell surface in agreement with a lack of detectability of redistributed PABP. However, cells undergoing cell adhesion for 60 min exhibited a well-defined puromycin staining in dot-like structures at the cell membrane, which also colocalized with ADAM15 and PABP signals, respectively (Fig 6), suggesting that an ongoing mRNA translation not only occurs at the cell membrane during the adhesion process, but also compartmentalizes within microdomains of ADAM15 and PABP expression. All stainings are shown exemplified for human chondrocytes, but synovial fibroblasts exhibited identical staining patterns (data not shown).

**Fig 7:**
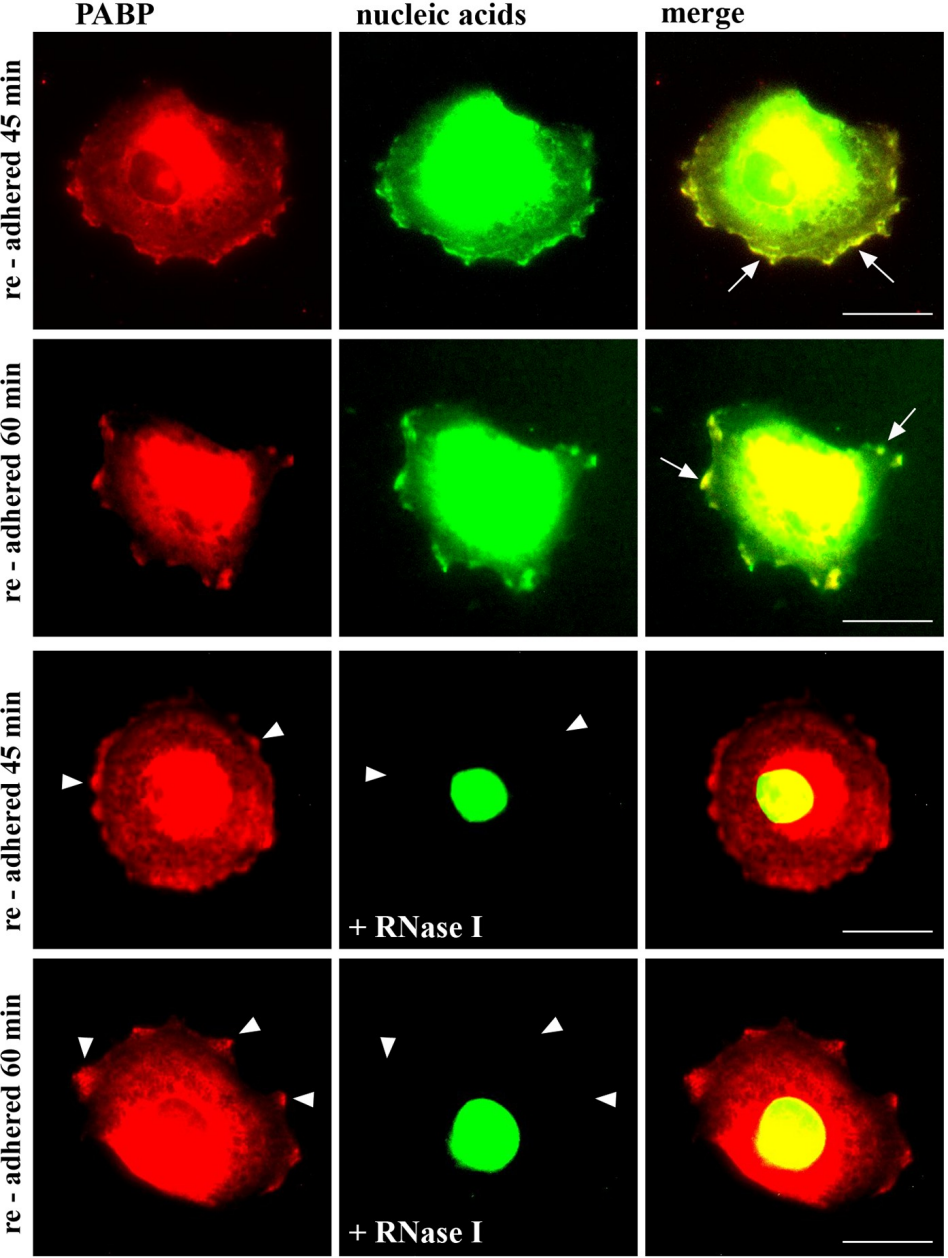
PABP colocalizes with RNA at the cell membrane. Confocal microscopy of synovial fibroblasts that were re-adhered for 45 and 60 min and stained with PABP antibodies and Alexa 594 (red) secondary antibodies and SYTO RNASelect Green, revealing colocalization of PABP with nucleic acids (white arrows). Prior to staining with SYTO Green, cells were treated with RNase (5 U/ml I) for 20 min. White arrow-heads indicate areas of no overlap due to RNase treatment. Objective x40, size bar = 20 μm.

Given that mRNA translation occurs at the cell membrane, we next analyzed, whether PABP colocalizes with RNA at the cell membrane using the nucleic acid fluorophore SYTO RNASelect Green for a direct detection of RNA. Synovial fibroblasts were re-adhered for 45 and 60 min and stained with anti-PABP antibodies and SYTO RNASelect Green. Consistent with the results shown in Fig 4, PABP redistributed to the cell membrane and accumulated in larger areas over the entire cell surface after 45 min of cell adhesion, whereas prolonged adhesion for 60 min resulted in a concentration of PABP detectability to more circumscribed patch-like areas at the surface (Fig 7). The same distribution pattern was observed for the nucleic acid staining after cell adhesion. However, incubation of cells with RNase I (5U/ml) for 20 min eliminated all cytoplasmic as well as membrane-localized SYTO RNASelect Green staining (Fig 7).

Taken together, our data demonstrate ongoing mRNA translation associated with PABP and ADAM15 colocalization at the cell membrane upon triggering of cell adhesion.

### Puromycin-terminated proteins are detected in plasma membrane preparations of ADAM15-expressing cells

**Fig 8:**
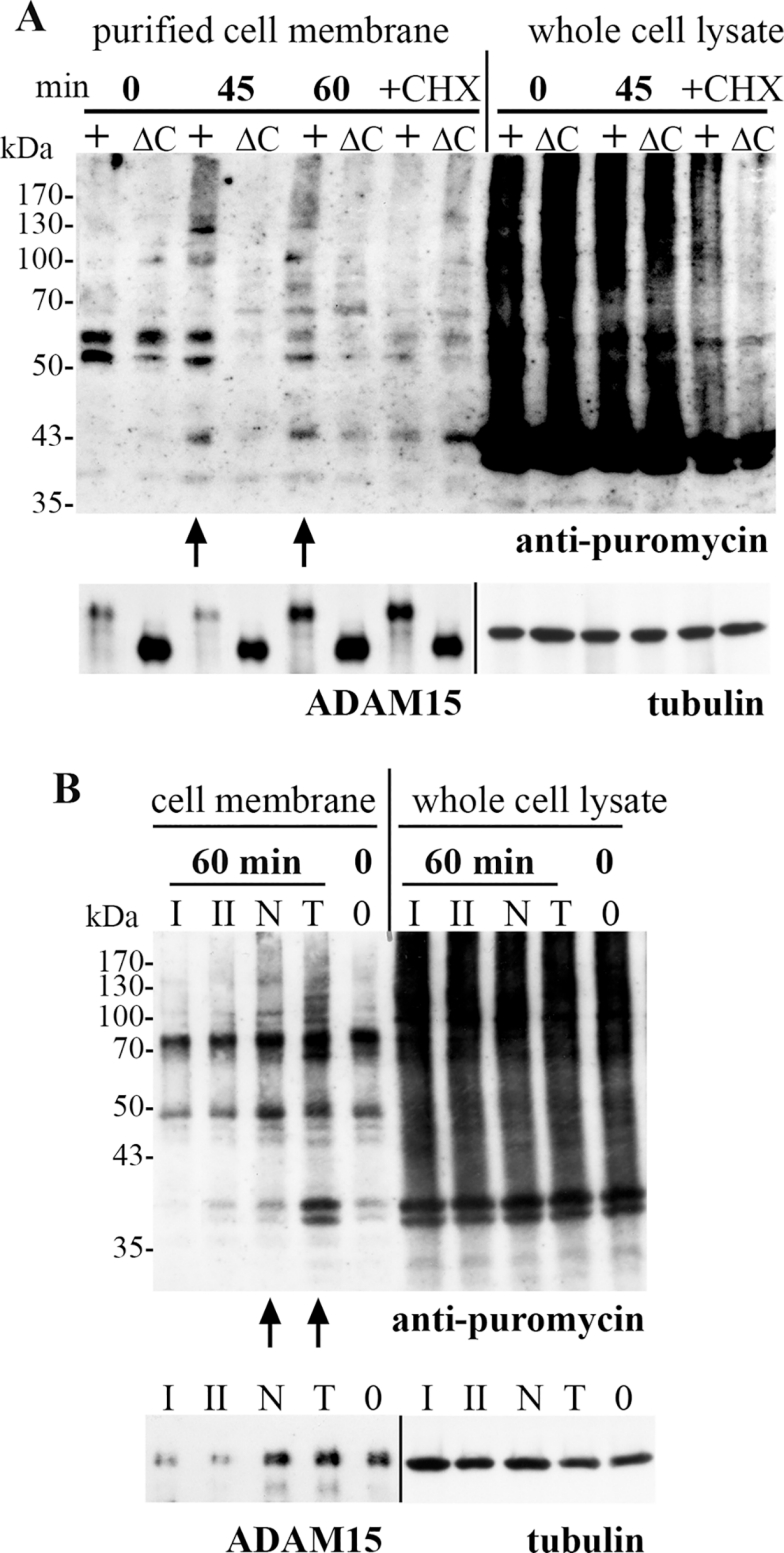
Puromycin-terminated proteins are detected at the cell membrane in the presence of ADAM15. (A) chondrocytes transfected with full-length ADAM15 (+) and the deletion mutant of ADAM15 lacking the cytoplasmic domain (ΔC) were detached and re-adhered for 45 and 60 min, fed with puromycin, crosslinked with PFA and cell surface biotinylated. The streptavidin purified membrane proteins as well as whole cell lysates were analyzed by immunoblotting using anti-puromycin IgG, showing markedly more puromycin-conjugated protein fragments in the plasma membrane of ADAM15-transfected cells after 45 and 60 minutes of adhesion (marked by arrows). Additional co-incubation with cycloheximide (CHX), showing nearly complete inhibition of protein translation at the plasma membrane. The blots were cut in half and reprobed using either ADAM15 or tubulin antibodies to check for the expression of full-length and deletion mutant ADAM15 or for equal loading, respectively. (B) HeLa cells were subjected to the same experimental procedures as described in (A), but prior to that the ADAM15 protein expression was downregulated by two specific siRNAs (I and II) and compared to a nonspecific control siRNA (N) or transfection reagent (T), showing stronger staining of puromycin protein conjugates in ADAM15 expressing HeLa cells after 60 min adhesion when compared to ADAM15 silenced cells or normally grown cells (0 min). One half of the membrane was reprobed using anti ADAM15 IgG showing a silencing efficiency of ~80%, the other half using tubulin IgG as a loading control.

To validate the results obtained by immunocytological stainings, puromycin-terminated translated protein fragments were analyzed by immunoblotting of biotinylated cell membrane preparations. Full-length ADAM15 or the ADAM15-mutant lacking the cytoplasmic domain (ΔC) transfected cells were fed puromycin for 10 min, and subjected to re-adhesion for 45 and 60 minutes. The cell surface was crosslinked with PFA, biotinylated and streptavidin-affinity purified cell membrane proteins analyzed by Western Blotting using anti-puromycin antibodies. The cell membranes of normally grown, adherent cells exhibited a moderate staining for puromycylated proteins with the exception of two protein fragments at ~ 50 kDa. However, after re-adhesion for 45 and 60 min the cell membrane of ADAM15- cells revealed significantly increased signals of puromycin-conjugated proteins in the range of 50 kDa to > 300 kDa and one prominent band at 43 kDa, which could not be detected in ADAM15 Δcyto-cells (Fig 8A). As expected, the whole cell lysates of ADAM15- and ADAM15Δcyto- cells showed puromycin incorporation into newly synthesized proteins to a markedly higher degree than that found in the isolated cell membrane preparations. Thus, the puromycin-conjugated proteins migrating as a strong smear throughout the whole lane (Fig 8A), reflect the dominance of mRNA translation in the whole cell compared to that compartmentalized to the cell membrane. Additional co-treatment with the translation inhibitor cycloheximide revealed markedly decreased signals of puromycin-conjugated proteins in both the isolated cell membranes and whole cell lysates of ADAM15- and ADAM15Δcyto- cells in comparison to puromycin incubation alone.

Furthermore, the occurrence of mRNA translation at the cell membrane in dependency of ADAM15 was also detected in human HeLa cells using immunological detection of puromycin-conjugated proteins in cell membranes after ADAM15 down regulation. ADAM15 protein expression was down regulated by ADAM15 specific siRNA I and II for 48 hours, cells subjected to re-adhesion for 60 min, puromycin fed, PFA crosslinked, and the biotinylated cell surface isolated. As shown above, puromycin-terminated proteins could be detected as a smear throughout the whole lanes in whole cell lysates irrespective of the protein expression level of ADAM15 and/or the re-adhesion stimulus applied (Fig 8B). Also, generally considerably less puromycin signals were detected in the cell membrane preparations of normally grown cells or re-adhered for 60 min. However, the signals of puromycylated proteins were even further reduced upon specific silencing of ADAM15 after 60 min of re-adhesion as compared to the normally grown cell state (0 min, Fig 8B), clearly showing that the specific downregulation of ADAM15 or deletion of its cytoplasmic tail impacts an ongoing mRNA translation at the cell membrane.

### Puromycin-terminated proteins colocalize with focal adhesion kinase at the cell periphery during cell adhesion

**Fig 9:**
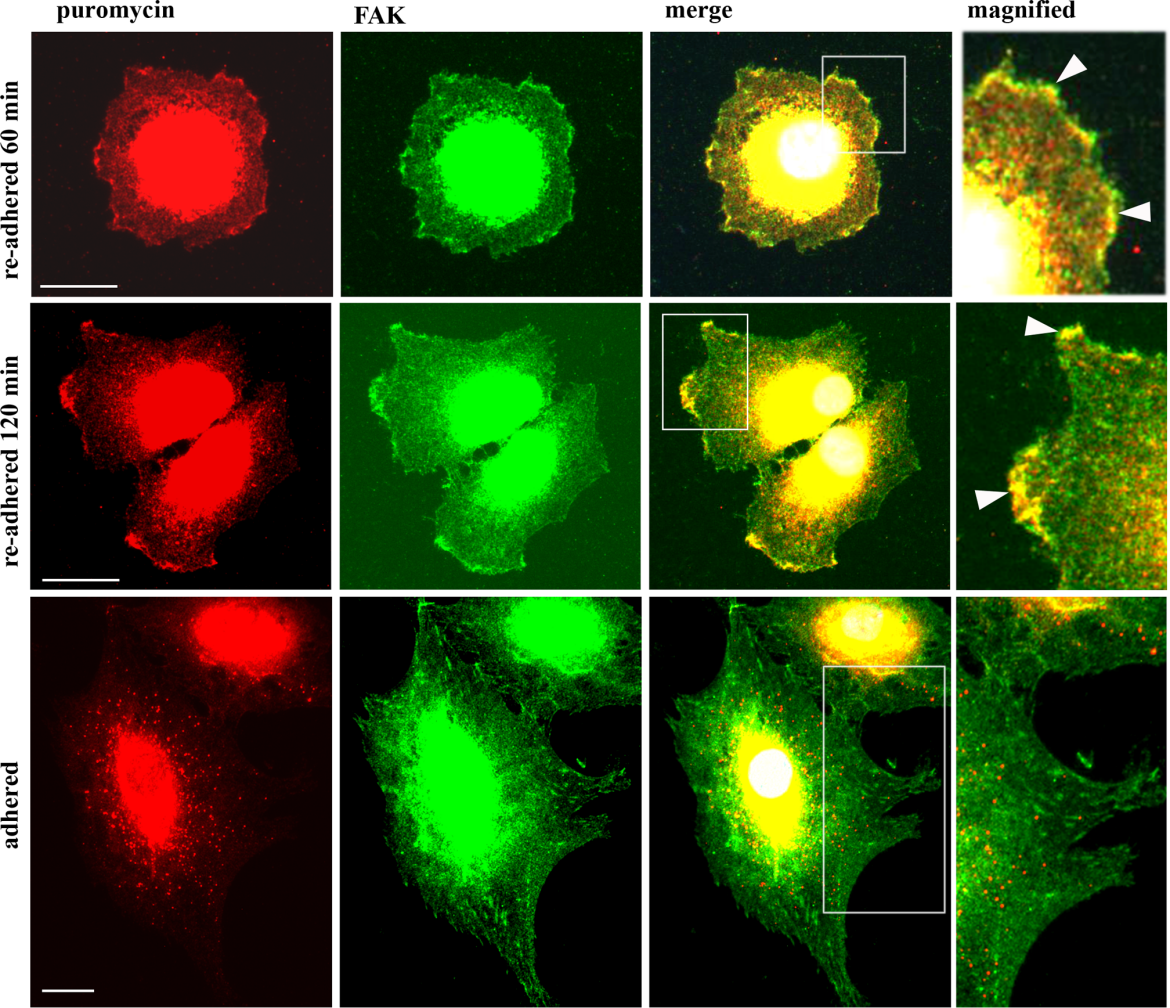
Puromycin-terminated proteins colocalize with focal adhesion kinase (FAK). Confocal microscopy of synovial fibroblasts that were re-adhered for 60 and 120 min, fed with puromycin, double stained using anti-puromycin and FAK antibodies, followed by visualization with Alexa 488 and 594 secondary antibodies, showing colocalization of puromycylated proteins with FAK at the cell membrane during adhesion only (white arrows in magnified area). Objective 40x, size bar = 20 μm.

Next, we investigated, whether the immunostaining signals derived from puromycylated proteins and ADAM15/PABP complexes at the cell membrane colocalize with FAK. FAK, a marker for focal contacts, has been shown previously to interact directly with ADAM15, and both proteins colocalize in focal contacts in adherent cells [25]. Synovial fibroblasts were re-adhered for 1 hour, then fed with puromycin and processed for double immunofluorescence stainings using anti-FAK and anti-puromycin antibodies as described above. Staining of FAK is detected in very distinct and fine dot- and/or dash-like staining patterns at the surface of cells that appear still very round in an early phase of the adhesion process. The puromycin staining showed the same staining pattern and revealed colocalization with FAK (Fig 9). When the cells started to spread, the FAK- as well as puromycin-stainings change into a patchy-like appearance and become more confined to certain areas of the cell membrane. After completed adhesion and cell spreading, puromycylated protein complexes were not detectable anymore at the cell membrane, but remained clearly present in the cytoplasm, especially in the region around the nucleus (Fig 9). In contrast, FAK staining revealed the typical staining pattern for focal adhesions at the cell membrane in spread out cells. These data clearly show, that the stimulation of cell adhesion results in the induction of an ongoing protein translation at the cell membrane, as evidenced by the detection of puromycylated proteins that disappears once adhesion is completed.

## Discussion

### Identification of PABP as a new ligand of ADAM15

A role of the transmembrane-anchored disintegrin metalloproteinase ADAM15 in integrin mediated adhesion to matrix molecules via modulation of downstream signaling in complex with focal adhesion kinase and c-src [6, 8, 25, 27] is well established. The present study provides substantial new evidence to suggest a yet unexplored function of ADAM15 in the control of ribosomal mRNA translation ongoing close to the cell membrane.

Thus, our studies demonstrate a specific and selective binding of ADAM15 to the proline-rich linker of PABP that connects the N-terminal RRM cluster and the C-terminal globular domain [14]. Several translation factors bind to the C-terminus of PABP, and especially to the PABC domain, such as the translation initiation factor eIF4B, PABP-interacting proteins Paip-1 and Paip-2, and eukaryotic release factor 3 (eRF3) and orchestrate the interaction of PABP with poly(A) RNA during translational activation in a so-called “closed loop model” [28]. However, no other endogenously transcribed ligands have been described so far to specifically interact with this PABP domain. Nevertheless, the PABP linker is a target for viral proteases like poliovirus 3C protease and its cleavage is instrumental in shutting off the poly(A) dependent host cell translation machinery during viral infection [29]. For ADAM15, on the other hand, the majority of its intracellular interactions have described to involve proteins containing SH2/SH3 (Src Homology) motifs. Thus, the cytoplasmic domain of ADAM15 can bind to ligands containing the–PXXP–core motif of SH2/SH3 containing proteins, like c-src, Grb2 or sorting nexin 9 and 33 [30], but also SH2/SH3 independent protein binding to ADAM15 has been described. Thus, a direct binding of its cytoplasmic domain to the C-terminus of FAK has been shown to enhance FAK-Src complex activation in response to apoptosis-inducing genotoxic stress, thereby reinforcing counter-regulatory survival pathways [25].

### Localization of ADAM15 binding to PABP at the cell membrane

In adherently growing chondrocytes or synovial fibroblasts ADAM15/PABP interaction is not detectable at the cell membrane by double immunofluorescence stainings. The strong signals for both proteins in the perinuclear cytoplasmic region, however, likely reflect their colocalization in the ER.

To address the question on the nature of a potential stimulus that could trigger an interaction between ADAM15 and PABP at the cell membrane, a reference to the functional role of ADAM15 in cell-cell and cell-matrix interactions turned out as very instructive: Thus, binding of its extracellular disintegrin domain via an Arg-Gly-Asp motif (RGD) to various integrin α and β chains has been demonstrated by protein binding studies in vitro [2, 3, 31, 32]. Moreover, it was shown in cell adhesion assays using integrin blocking antibodies or RGD-blocking peptides that ADAM15 confers an enhanced integrin-dependent cell binding to extracellular matrix proteins [6] and colocalizes with FAK in focal contacts in cells grown on extracellular matrix proteins, like collagen and fibronectin [25]. A complementary observation was provided by the immunohistochemical detection of PABP at the cell surface in about 20% of HeLa cells. The detected dot-like staining pattern associated with cells exhibiting a round shape changed into a dash-like appearance upon cell spreading, thereby resembling focal adhesion structures that emerge during focal contact formation. Accordingly, we developed a re-adhesion assay whereby cells were detached by EDTA and subsequently re-adhered to chamberslides. Using this stimulus, we detected colocalization of PABP with ADAM15 at a large proportion of the cell periphery. These findings were corroborated by immunoblotting studies of biotinylated cell membrane preparations, whereby cell surface recruited PABP is detected as early as after 30 min after induction of cell adhesion. Moreover, this redistribution of PABP to the cell membrane was clearly demonstrated to depend on ADAM15 expression, since cells transfected with an ADAM15 deletion mutant that lacks the cytoplasmic tail show considerably reduced levels of PABP at the cell surface upon triggering adhesion.

As reported in the literature, the majority of PABP displays a predominant diffuse cytoplasmic localization in cells at steady state [14], but its occurrence has also been demonstrated in the nucleus or in stress granules. In response to cellular stress the subcellular localization of PABP can change drastically either by accumulation in cytoplasmic foci including those associated with mRNA storage and localized translation or in the nucleus [33]. Thus, PABP is a major component of cytoplasmic stress granules induced by cellular stress and storage in these structures is associated with an emergency reaction to control cell fate decisions [34]. Additionally, upon UV exposure PABP has been shown to shuttle into the nucleus which is accompanied by a reduced protein synthesis [35]. A yeast PABP was described to shuttle quickly between the cytoplasm and the nucleus by binding to the protein export factor Xpo1/Crm1, thereby functioning as an export protein for poly(A) RNA [36]. But also, the localization of PABP at the cell surface was observed, as demonstrated at the leading edge (or lamellipodium) of migrating cells [37]. Moreover, the interaction of PABP and paxillin, a protein of the focal adhesion complex, was demonstrated as a critical component for an efficient transport of PABP from the nucleus to the cytoplasm and the subsequent remodeling of focal adhesions during cell migration [38].

Collectively, our data clearly reveal an occurrence of PABP at the cell membrane that is induced by cell adhesion and also crucially dependent on the expression of ADAM15. The detectability of PABP recruitment to the cell surface during cell adhesion as a conserved phenomenon in primary cells as well in all immortalized cell lines of the present study suggests that it might represent an advantageous general mechanism ensuring the local synthesis of specific proteins needed for cell adhesion.

### De novo protein translation colocalizing with ADAM15 and PABP in focal contacts

Our investigations show that de novo protein synthesis occurring at the cell membrane is dependent on ADAM15 as well as PABP in that compartment. These results were obtained by the application of the so-called Surface Sensing of Translation (SUnSET) technology [26] that uses immunostaining to detect puromycin-terminated protein fragments as a specific signal for ongoing ribosomal mRNA translation. Contrary to adherently growing cells that exhibited protein neosynthesis associated signals only in the cytoplasm, cells undergoing re-adhesion displayed cell surface linked puromycylated protein fragments in colocalization with either ADAM15 or PABP. The obtained fluorescence signal patterns present as dot-like and/or dash-like structures strongly resembling focal adhesion structures that are shaped in the process of focal contact formation.

To confirm the nature of these structures, we chose FAK as a marker that is specifically recruited to focal adhesions where it plays a pivotal role in their formation as well as in concert with integrin ligation cell survival downstream signaling [39]. The colocalization of FAK with the puromycin conjugates at the cell membrane of adhering cells provides clear evidence for an ongoing mRNA-translation in focal contacts formed during adhesion. ADAM15 has earlier been shown to colocalize with FAK in such focal contacts in adherently grown cells at steady state [25]. These results are consistent with published data that imply various proteins of focal adhesions as components of local mRNA translation at the cell membrane. Thus, the recruitment of poly(A) mRNA and ribosomes to focal adhesions in response to integrin stimulation was demonstrated [40]. Moreover, binding of the PABP interacting translation factors eIFG4G and eIF4E with vinculin that links integrin-mediated focal adhesion junctions to the actin cytoskeleton, was detected in focal adhesions in cells grown on fibronectin [41]. Also, colocalization of PABP with paxillin recruited to focal adhesions in protrusions of migrating cells [42] demonstrates the occurrence of mRNA translation at the cell membrane in lamellipodia [37], although in this case an explicit association with structures of focal contacts was not shown. Moreover, the occurrence of PABP in the so-called integrin adhesome was identified by mass spectrometry during integrin adhesion complex (IAC) assembly. These IACs are formed by integrin receptor activation at the cell membrane, thereby transducing adhesion dependent signaling [43]. Along that line, vinculin mediates the recruitment of raver1, an RNA binding protein of the heterogeneous nuclear ribonucleotide proteins (RNP) that harbors three RNA recognition motifs at the N-terminus, together with its cargo mRNA to focal adhesions, suggesting the local production of components of adhesion complexes [44]. Also, the recruitment of other families of RNA-binding proteins, the Sm proteins and hnRNPs, to the so-called “spreading initiation centers (SICs)”, which are structures that assemble early during the initial cell spreading and precede the formation of focal adhesions, has been shown to colocalize with RNA at the cell membrane [45]. As the focus of our studies was to elucidate for the first time a functional role of a disintegrin-metalloproteinase in protein translation via PABP interaction, the uncovering of the precise timely sequence of the complex events involved in the formation of focal adhesions via intermediate steps, including SICs, and their impact on ADAM15/PABP interaction will remain to be addressed by future investigations.

Collectively, our data show a colocalization of puromycin-terminated proteins with PABP and ADAM15 in focal contacts as a local membrane compartment of ongoing mRNA translation that is triggered by cell adhesion. Thus, a specific down regulation of ADAM15 in HeLa cells and/or the deletion of the cytoplasmic tail of ADAM15 yielded considerably lower signals for puromycylated proteins in the cell membrane after induction of adhesion, revealing a new function of ADAM15 that is present only for a short time in the cell cycle. Two hypothetical scenarios for ADAM15 that might occur simultaneously during adhesion might be envisioned: 1. ADAM15 could serve as an anchor protein for PABP with its bound mRNA thereby facilitating the interaction with the protein translation complex and 2. it might contribute to capture the translation machinery to the cell membrane during adhesion. As has been reviewed elsewhere [46], for protein synthesis to be spatially restricted a complex interplay between transport ribonuclein particles and repressors of translation keeping the mRNAs sequestered from the biosynthetic machinery until getting derepressed either by prelocalized interacting proteins or a specific signaling trigger in their final destination is required. As our studies did not focus on an involvement of ADAM15 in mRNA transport to the cell membrane, an elucidation of these hypothetical connections goes far beyond the scope of the present studies and remains to be unravelled by future investigations. However, our study provides initial evidence for a potential involvement of ADAM15 in a respective targeting control mechanism of mRNA translation during cell adhesion.

## Materials and methods

### Antibodies and reagents

Several antibodies directed against the extracellular part ADAM15 were used: a goat polyclonal (# AF935) and a mouse monoclonal (# mab945) from R&D Systems. Rabbit anti-ADAM15 (cytoplasmic domain, # ab84834), rabbit anti-PABP (# ab21060), rabbit anti-tubulin (# ab134185) and rabbit anti-calnexin antibody (# ab22595) from Abcam. Mouse anti-CD25 (# 174–820) from Ancell-Enzo Life Sciences GmbH. Mouse anti-puromycin (# MABE343, clone 12D10), mouse anti-myc antibody, (# 05–724), rabbit anti-alpha5 integrin (# AB1921) from Merck Millipore. Rabbit anti-FAK (# AHO0502) was from Invitrogen. Anti-Glutathion-S-Transferase peroxidase conjugate (# A7340) from Sigma-Aldrich.

### Generation of permanent cell lines transfected with ADAM15

Full-length ADAM15 and mutants lacking the cytoplasmic domain were cloned as described previously [6, 28]. Cells that were transfected with the empty pExchange-1 vector served as a control. The transfection and selection procedures were performed as described [6]. The chondrocyte cell line T/C28a4 was kindly provided by Dr. M.B. Goldring (Hospital for Special Surgery, NY, NY), [47].

### Isolation of human osteoarthritic (OA) chondrocytes and synovial fibroblasts

OA chondrocytes and RA synovial fibroblasts were isolated by sequential pronase and collagenase digestion. Cartilage was cut into small pieces and digested with pronase (1mg/ml PBS) for 30 min at 37°C and then with bacterial collagenase (1.5 mg/ml PBS) for 16–24 h. The human specimens were derived from the hip or knee joints of patients who had undergone endoprosthetic joint replacement for severe osteoarthritis. The tissues samples were obtained with written consent from the patients (an ethics approval was provided by the Institutional Review Board of the University Hospital Frankfurt). The tissue samples were kindly provided by Dr. Adolf, Orthopedic University Hospital Friedrichsheim at the Goethe University Frankfurt am Main, Germany.

### Cell culture

The chondrocyte cell line transfected with full-length ADAM15, the deletion mutant without the cytoplasmic domain, or vector control were kept in DMEM (Dulbecco’s modified Eagle’s medium) supplemented with 10% heat-inactivated fetal calf serum (FCS). For all subsequent tests cells were grown to subconfluency to a cell density of about 4 x 10^6^ cells/75 cm^2^ tissue culture flask. OA chondrocytes were cultivated in Ham12/DMEM plus 10% FCS from cell passage 4–6. Synovial fibroblasts were cultivated in DMEM plus 10% FCS from cell passage 4–6. Penicillin (50 U/ml) and streptomycin (50 mg/ml) were added to all media. All tissue culture reagents were from Gibco/Invitrogen.

### Preparation of cell lysates and Western Blotting

Transfected cells were washed with ice-cold PBS, scraped off the tissue culture flask and lysed in RIPA buffer for 1 hour at 4°C (150mM NaCl, 50mM Tris/HCl, pH 7.2, 1% Nonidet P40, 1% Triton X-100, 0.25% sodium deoxycholate, 5mM EDTA containing a proteinase inhibitor cocktail (according to the supplier´s formulation and instruction: 1 tablet/10 ml, Roche Diagnostics) and phosphatase inhibitor cocktail I and II (10 μl/1ml lysis buffer, Sigma-Aldrich). After centrifugation for 5 min at 4°C, protein concentration was determined using BCA reagent (Pierce). Samples were separated by 10% SDS/PAGE, transferred to nitrocellulose filter and incubated with the respective antibodies overnight at 4°C. Blots were incubated with appropriate HRP-conjugated antibodies (1:5000, Dako, Hamburg) and developed using the ECL Plus Western Blotting Detection System (Amersham GE Healthcare).

### Preparation of cell lysates and co-immunoprecipitation

Cells transfected with vector control and full-length ADAM15 were washed with ice-cold PBS, scraped off the tissue culture flask and lysed in 10 mM HEPES, pH 7.0, 150 mM NaCl, 5mM EDTA, 1% Triton X-100 and complete proteinase inhibitor cocktail (Roche Diagnostics; 10 μl/ml lysis buffer) as described above for 1 hour at 4°C. Protein concentration was determined and diluted to a concentration of 4mg/ml. For co-immunoprecipitation experiments, the mouse monoclonal anti-ADAM15, the goat polyclonal anti-ADAM15, that co-immunoprecipitated identical bands, were incubated with the freshly prepared cell lysates and Protein G conjugated agarose beads (Pierce) under constant agitation for 90 minutes at 4°C. The beads were washed 4 times with lysis buffer, boiled in sample buffer (125 mM Tris/HCl, pH 6.8, 4% SDS, 40% Glycerol, 10% β-mercaptoethanol, 20 μg/ml bromophenol blue) for 5 minutes at 95°C and subjected to SDS/PAGE. Subsequent Western Blotting was performed as described above.

### MALDI-TOF

Excised proteins from a SDS gel were identified by MALDI-TOF on a Bruker Reflex IV mass spectrometer by Dr. Stefan Müller, Proteomics Facility, Centre of Molecular Medicine (ZBA), University of Cologne, Germany, and analyzed using the MASCOT PMF program (Matrix Science, London, UK).

### Generation of recombinant domains of ADAM15 and PABP

All recombinant protein domains were cloned into the BamHI/NotI site of pGEX 3P (Amersham GE Healthcare, Munich, Germany) and expressed as a GST (glutathione S-transferase) fusion proteins in Rosetta(DE3)pLysS BL21 E.coli (Merck Millipore, Darmstadt, Germany). The cytoplasmic domain of ADAM15 (amino acids 717–814) was cloned as described previously (30). A myc-tag was attached to the C-terminus. The various PABP domains were amplified by PCR using a PABP vector (OriGene) as template and cloned using the primer combinations listed under mammalian-two hybrid. The correctness of the amplified nucleotide sequence was confirmed by sequencing. Protein expression was induced using 1mM IPTG for 3h at room temperature and the harvested E.coli were lysed with Bug Buster protein extraction reagent (5 ml/ 1g bacterial pellet) and benzonase nuclease (1 U/ml) according to the supplier’s instructions (Merck). The supernatant containing the GST-tagged fusion proteins were incubated with GST-sepharose (Amersham) for 30 minutes at room temperature, thoroughly washed with PBS (150 mM NaCl, pH 7.4, 1mM KH_2_PO_4_, 3 mM Na_2_HPO_4_) containing 1% Triton X-100. The GST-fusion proteins were eluted with 10mM reduced glutathione in 50 mM Tris/HCl, pH 8.0, containing a protease inhibitor cocktail (Roche Diagnostics). In order to remove the GST-tag, the GST-sepharose with the bound GST fusion proteins was washed thrice with PBS/Triton X-100 and equilibrated by washing twice with protease cleavage buffer (25 mM Tris/HCl pH 8.0, 150 mM NaCl, 14 mM β-mercaptoethanol). After treatment with Precission Protease for 24 h at 4°C according to the manufacturer’s instructions (Amersham), the supernatant containing the proteins was collected by centrifugation. After thorough washes with PBS/Triton X-100, GST was then eluted with the 10mM reduced glutathione buffer. Protein concentration was determined using BCA (bicinchoninic acid) reagent (Pierce).

### Protein binding assays

A 96-well plate was coated with the cytoplasmic domain of ADAM15 (100 ng/100 μl in PBS) blocked with 1% BSA in PBS for 1h, and incubated with the GST-tagged PABP domains (500 nM) for 1h at room temperature. After three washes with PBS/ 1% Triton X-100, wells were incubated with HRP-conjugated anti-GST antibodies (1:500; Sigma-Aldrich) for 1h, developed with 100μl ABTS solution (Roche, Mannheim, Germany) and the optical densitometry (OD) read at 405 nm.

### Mammalian-two hybrid

The interaction of ADAM15 with PABP was analyzed using the mammalian two-hybrid assay kit from Stratagene. The cytoplasmic tail of ADAM15 (amino acids 717–814) was cloned into the BamHI/NotI site of the pCMV-AD prey vector using a full-length ADAM15 plasmid as template for PCR amplification as described in detail [25]. The PABP domains 1–192, 392–636, 392–553, 392–470, and 553–636 were cloned accordingly into the pCMV-BD bait vector using reverse transcribed cDNA from chondrocytes as template and following primers: 1–192: 5’ TAGGATCCGCTATGAACCCCAGTGCCCCCA 3’ and 5’ TTGCGGCCGCCTATTCTTTTGCCCTAGCTCCAAGTT 3’; 392–636: 5’ TGGATCCATGCCCAACCCTGTAATCAACCCCTA 3’ and 5’ TGCGGCCGCCTAAACAGTTGGAACACCGGTGGCA 3’; 392–553: 5’ TGGATCCATGCCCAACCCTGTAATCAACCCCTA 3’ and 5’ TGCGGCCGCCTAGGCAGATGCCAACATGGAAGCA 3’; 392–470: 5’ TGGATCCATGCCCAACCCTGTAATCAACCCCTA 3’ and 5’ TGCGGCCGCCTAAGAAGCTGGTCTCATAGTACTAAATGG 3’; 553–636: 5’ TAGGATCCATGTCTGCCCCTCCTCAAGAGCAAA 3’ and 5’ TGCGGCCGCCTAAACAGTTGGAACACCGGTGGCA 3’. HEK 293 cells (1x10^4^) were seeded into a 96 well, grown for 24 hours and co-transfected with the bait, prey vector (10ng each), the firefly luciferase reporter (250 ng) and 5ng renilla luciferase plasmid (pRL-TK vector, #E2241, Promega) to control for equal transfection rate using JetPei according to the supplier’s instructions (Biomol). The control plasmids pBD-p53 and pAD-TRAF (included in the kit), whose expressed proteins do not interact in vivo, were also transfected and served as a negative control. 48 h after transfection the expression of the luciferase was measured using the Dual-Luciferase Reporter Assay System (Promega) with the Mithras LB 940 plate reader (Berthold). Quadruplicate wells were transfected and the assay performed at least five times.

### Isolation of plasma membrane by density gradient ultra-centrifugation

Cells grown in petri dishes were scraped off and harvested by centrifugation at 1900 rpm for 5 min. The cell pellet was resuspended in 1.6 ml HBS (50 mM HEPES, pH 7.0, 150 mM NaCl, 1.5 mM Na_2_HPO_4_ x 2 H_2_O, containing proteinase inhibitors), lysed by ultrasonication on ice (4x 20 s, 7 cycles, 50% intensity) and centrifuged at 450 x g for 5 min. The supernatant (0.4 ml) was carefully pipetted onto a sucrose-gradient, consisting of various concentrated sucrose solutions: 1.6 ml—60%, 1.6 ml—38%, 1.28 ml—15% Sucrose (w/v), in 20 mM Tris/HCl, pH 7.4. The gradient was subjected to ultracentrifugation in a swinging bucket rotor without brake for 1h at 112000 xg. The layer between the 60% und 38% sucrose was collected with a syringe needle, diluted with 20 mM Tris/HCl, pH 7.4 and centrifuged for 2.5 h at 186000 x g. The pellet containing membrane proteins was solubilized with PBS/1% Triton X-100 plus protease inhibitors overnight at 4°C, then ultrasonicated for 5 min and centrifuged for 15 min at 10000 x g. The supernatant contained the Triton soluble fraction, which then was analyzed by Western blotting.

### Isolation of plasma membrane by cell surface biotinylation

ADAM15 transfected cells and HeLa cells were grown to subconfluency in petri dishes, and after washing twice with PBS incubated with the membrane impermeable EZ-Link Sulfo-NHS-LC-LC-Biotin from Pierce (0.1 mg/ml in PBS, pH 8.0, 0.9 mM CaCl_2_, 0.5 mM MgCl_2_) for 15 min at room temperature. After washing twice with PBS, cells were fixed with 1% PFA in PBS for 5 min at room temperature, and then quenched with 2.5 M glycin for 5 min. Cells were scraped off and harvested by centrifugation at 3000 xg for 5 min at 4°C and the cell pellet washed twice with PBS and lysed in 10 mM HEPES, pH 7.0, 150 mM NaCl, 5mM EDTA containing 1% Triton X-100 and complete proteinase inhibitor cocktail (Roche Diagnostics) and ultrasonicated on ice. Cell debris was removed by centrifugation at 13.000 rpm for 5 min, and the supernatant incubated with streptavidin conjugated magnetic beads (10 μl beads / 500 μg cell lysate; Thermo Fisher) and 1U benzonase nuclease (Merck) under rotation for 2h at room temperature. The magnetic beads were separated using a magnet, washed thrice with PBS/0.1% Triton X-100, and boiled in 20 mM Tris/HCl, pH 6.8, 0.5% SDS, 10% Glycerin v/v, 0,1% bromophenol blue w/v, 1% β-Mercaptoethanol for 15 min at 99°C to revert the PFA crosslinked protein complexes.

### Re-adhesion assay

Human primary OA chondrocytes or synovial fibroblasts grown overnight were washed twice with PBS, gently detached using 5 mM EDTA in PBS, and seeded into chamberslides (Falcon, Thermo Fisher) for subsequent immunofluorescence stainings or into petri dishes for cell surface biotinylation. Chamberslides and petri dishes were coated with bovine fibronectin (2 μg/ml, Sigma-Aldrich) for 1h at 37°C or at 4°C overnight.

### Immunofluorescence

Human osteoarthritic chondrocytes, the synovial fibroblasts, and HeLa cells (10^5^) were grown on chamberslides for 24 h, fixed with 4% Paraformaldehyde (PFA) in PBS for 5 min and blocked with 1% BSA in PBS, 0.1% Triton X-100 for 1h. After staining with goat anti-ADAM15 (1:100) overnight, mouse anti PABP (1:100) or mouse anti-Puromycin (1:10.000) for 1h, cells were washed thrice in PBS, 0.1% Triton X-100 and incubated with Alexa Fluor conjugated secondary antibodies: goat anti-mouse 488, donkey anti-goat 488, goat anti-mouse 594 (1:500, Invitrogen), washed, nuclei counterstained with DAPI (4′,6-diamidino-2-phenylindole, dilution: 1:500, Invitrogen) and mounted using Fluorescent Mounting Medium (Dako). For the detection of RNA, cells were incubated for 10 min with 100 μg/ml SYTO™ RNASelect™ Green Fluorescent cell Stain (Invitrogen). To induce colocalization of ADAM15 with PABP, cells that were grown adherently for 24 h were washed twice with PBS and detached from the tissue culture vessels with EDTA in PBS. Reattachment was allowed for 60–120 min in DMEM plus 10% FCS, followed by fixation in 4% PFA in PBS, pH 7.4. Fluorescence images were analyzed and recorded using a LSM 510 Meta Laser Scanning Confocal Microscope (Carl Zeiss) with the included software from Zeiss. After recording, digital images were processed and adjusted for brightness and contrast using the ImageJ 1.47 program. All fluorescence images were taken under identical conditions.

### Staining of puromycin-terminated proteins

Primary human chondrocytes and synovial fibroblasts (1x10^3^) were grown overnight on chamberslides or were allowed to re-adhere for 1h in DMEM / 10% FCS. After re-adhesion the medium was replaced by DMEM containing puromycin (50 μg/ml) and cycloheximide (100 μg/ml) to block the dissociation of polypeptide-puromycin conjugates from ribosomes for 5 min at 37°C. Cells were washed with ice cold PBS / cycloheximide (100 μg/ml) and permeabilized on ice for 1 min with PBS/cycloheximide containing 0.025% saponin to wash out free puromycin and to reduce background staining in immunohistochemical analyses. After washing once with ice cold PBS/cycloheximide solution, cells were immediately fixed with 4% PFA in PBS, and stained with mouse anti-puromycin (1:10.000) and rabbit anti-PABP antibodies (1:100) or mouse anti-Puromycin and goat anti-ADAM15 (1:50) antibodies for 1h and visualized with Alexa 488 or Alexa 594 conjugated secondary antibodies (1:500, Invitrogen), as described in the section immunofluorescence.
